# TOMM40/FADS2 Expression Ratio Predicts the Sensitivity to mTOR Inhibitors in Triple‐Negative Breast Cancer

**DOI:** 10.1002/cam4.72089

**Published:** 2026-07-18

**Authors:** Salman Mohamed Farah, Jing‐Quan Zheng, Leon Tsung‐Ju Lee, Hsiao‐Wei Lu, Wen‐Ke Wang, Chia‐Hao Kuei, Hui‐Yu Lin, Hui‐Wen Chiu, Yuan‐Feng Lin

**Affiliations:** ^1^ International Ph.D. Program in Medicine, College of Medicine Taipei Medical University Taipei Taiwan; ^2^ Graduate Institute of Clinical Medicine, College of Medicine Taipei Medical University Taipei Taiwan; ^3^ Division of Pulmonary Medicine, Department of Internal Medicine, Shuang ho Hospital Taipei Medical University New Taipei City Taiwan; ^4^ Division of Pulmonary Medicine, Department of Internal Medicine, School of Medicine, College of Medicine Taipei Medical University Taipei Taiwan; ^5^ Department of Dermatology, School of Medicine, College of Medicine Taipei Medical University Taipei Taiwan; ^6^ Department of Dermatology Taipei Medical University Hospital Taipei Taiwan; ^7^ Department of Otolaryngology Head and Neck Surgery, Shuang ho Hospital Taipei Medical University New Taipei City Taiwan; ^8^ Department of Otolaryngology, School of Medicine, College of Medicine Taipei Medical University Taipei Taiwan; ^9^ Department of Surgery Taipei Medical University Hospital, Taipei Medical University Taipei Taiwan; ^10^ School of Medicine Fu‐Jen Catholic University New Taipei City Taiwan; ^11^ Division of Urology, Department of Surgery, Cardinal Tien Hospital Fu‐Jen Catholic University New Taipei City Taiwan; ^12^ Division of Breast Surgery and General Surgery, Department of Surgery, Cardinal Tien Hospital Fu‐Jen Catholic University New Taipei City Taiwan; ^13^ Department of Medical Research, Shuang ho Hospital Taipei Medical University New Taipei City Taiwan; ^14^ TMU Research Center of Urology and Kidney Taipei Medical University Taipei Taiwan; ^15^ Cell Physiology and Molecular Image Research Center, Wan Fang Hospital Taipei Medical University Taipei Taiwan

**Keywords:** FADS2, mTOR inhibitor, mTORC1, TOMM40, triple–negative breast cancer

## Abstract

**Background:**

Triple‐negative breast cancer (TNBC) is a subtype of breast cancer that lacks the expression of estrogen receptor (ER), progesterone receptor (PR), and human epidermal growth factor receptor‐2 (HER2). There is a lack of predictive biomarkers for the response of patients with TNBC to targeted therapies.

**Methods:**

Bioinformatics analysis was conducted to generate differentially expressed genes (DEGs) of the mammalian target of rapamycin complex 1 (MTORC1) gene set between normal tissues and primary tumors derived from TNBC patients using The Cancer Genome Atlas (TCGA) and Gene Expression Omnibus (GEO) databases. Cox regression analysis was performed to identify independent prognostic factors. Endogenous expression levels of the identified prognostic genes were detected in a panel of TNBC cell lines and breast cancer tissues using western blotting and immunohistochemistry (IHC).

**Results:**

Our findings revealed two prognostic genes: FADS2 and TOMM40. TOMM40 (HR = 2.243) was a risk factor and FADS2 was a protective factor (HR = 0.652). A higher TOMM40/FADS2 ratio is associated with poor outcomes in patients with TNBC. The TOMM40/FADS2 ratio was significantly (*p* < 0.05) associated with age, tumor size, lymph node metastasis, pathologic stage, and overall survival of patients with TNBC. Remarkably, the MTT cytotoxicity assay revealed that TNBC cells, which possess a higher TOMM40/FADS2 ratio than TNBC cells with a lower TOMM40/FADS2 ratio, are more sensitive to mammalian target of rapamycin (mTOR) inhibitor treatment.

**Conclusion:**

Our results provide a new therapeutic strategy using the TOMM40/FADS2 expression ratio to predict the cellular sensitivity to mTOR inhibitor treatment in TNBC.

## Graphical abstract

Our results provide a new therapeutic strategy using the TOMM40/FADS2 expression ratio to predict the cellular sensitivity to mTOR inhibitor treatment in TNBC. Our findings are of great importance in precision cancer medicine using the TOMM40/FADS2 expression ratio to discriminate patients with TNBC who would benefit from mTOR‐targeted therapy.

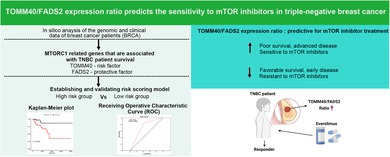

**Abbreviations**
Akt: Akt serine/threonine kinase
BRCA: breast cancer
BRG1: Brahma‐related gene 1
CRC: colorectal cancer
DEGs: differentially expressed genes
EC: endometrial cancer
EMT: epithelial to mesenchymal transition
ER: estrogen receptor
FADS2: D‐6‐fatty acid desaturase
FASN: fatty acid synthase
GEO: gene expression omnibus
HCC: hepatocellular carcinoma
HER2: human epidermal growth factor 2 receptor
IC50: inhibitory concentration 50%
IHC: immunohistochemistry
MAPK: mitogen‐activated protein kinase
MRTK: malignant rhabdoid tumor of the kidney
mTOR: mammalian target of rapamycin
mTORC1: mammalian target of rapamycin complex 1
mTORC2: mammalian target of rapamycin complex 2
mTRGs: mTORC1 pathway‐related genes
MTT: 3‐(4, 5‐dimethylthiazol‐2‐yl)‐2, 5‐diphenyl‐2H‐tetrazolium bromide
NSCLC: non‐small cell lung cancer
OS: overall survival
OvCa: ovarian cancer
PCOS: polycystic ovary syndrome
PI3K: phosphoinositide 3‐kinase
PIKK: PI3K‐related kinases
PR: progesterone receptor
PUFAs: polyunsaturated fatty acids
ROC: receiver operating characteristic
SCD: stearoyl‐CoA desaturase
SCD1: stearoyl‐CoA desaturase 1
SNPSs: single nucleotide polymorphisms
SREBP: Sterol regulatory element binding proteins
TCGA: The Cancer Genome Atlas
TNBC: triple‐negative breast cancer
TOM: translocase of the mitochondrial outer membrane (TOM)
TOMM40: Translocase of Outer Mitochondrial Membrane 40

## 1 Introduction

Triple‐negative breast cancer (TNBC) is a subtype of breast cancer that does not express estrogen receptor (ER), progesterone receptor (PR), or human epidermal growth factor receptor‐2 (HER2). TNBC is common in young women and is the most heterogeneous cancer, with a high recurrence rate and poor outcomes [1]. Chemotherapy is the current standard of care for TNBC patients. However, chemoresistance in neoadjuvant and adjuvant settings is challenging [2]. On the other hand, because of the negative expression of ER, PR, and HER2, targeted therapies used for other breast cancer subtypes do not work for TNBC [3]. Therefore, identification of useful biomarkers to predict the efficacy of targeted therapies for TNBC is urgently required.

Mammalian target of rapamycin (mTOR) is a serine/threonine protein kinase among PI3K‐related kinases (PIKK). mTOR is a core component of two protein complexes, mTOR complex 1 (mTORC1) and mTOR complex 2 (mTORC2), and its signaling plays a critical role in the proper regulation of cellular functions [4]. MTORC1 plays a central role in several cellular functions, including metabolic homeostasis, lipid and protein synthesis, proteasome assembly and autophagy [5]. Deregulated mTOR signaling has been reported to have implications in cancer and other diverse human diseases [6]. Previously, we reported that activation of Akt/mTOR signaling modulated metastatic progression [7] and chemoresistance in TNBC [8]. However, no study has investigated the prognostic significance of mTORC1 in TNBC.

Therefore, this study aimed to investigate the prognostic significance of MTORC1 gene set and to identify predictive biomarkers for the therapeutic effectiveness of mTOR inhibitors in TNBC. We obtained transcriptional data of normal tissues and primary tumors derived from TNBC patients from The Cancer Genome Atlas (TCGA) and Gene Expression Omnibus (GEO) databases, and used them to generate differentially expressed genes (DEGs) of the MTORC1 gene set from the Molecular Signatures Database. Univariate and multivariate Cox regression analyses of the overall survival of patients with TNBC were performed to identify genes that may have prognostic value in TNBC. The association between clinicopathological characteristics and prognostic genes was studied using the chi‐square test for independence. In vitro studies were used to detect the endogenous expression levels of prognostic genes and sensitivity/resistance of TNBC cells to mTOR inhibitor treatment. Our study showed that TNBC cells with high TOMM40/FADS2 expression ratios are more sensitive to mTOR inhibitor treatment.

## 2 Materials and Methods

### 2.1 Data Acquisition and Identification of Differentially Expressed Genes (DEGs) in the MTORC1 Gene Set

Gene expression profiles of normal mammary, TNBC, and non‐TNBC tissues were obtained from the Gene Expression Omnibus (GEO) dataset GSE58135 and The Cancer Genome Atlas (TCGA) database. The TCGA‐BRCA dataset contains IlluminaHiSeq pancan normalized gene expression data of 1218 tissues (solid tissue normal = 114, primary tissues = 1097 and metastatic = 7). Differential expression analysis was carried out with the solid tissue normal and primary tumors only. The classification of primary tissues into TNBC or Non‐TNBC was based on the status of ER, PR and HER2 receptors. All the 123 patients' data were used for analysis where applicable in the case of missing clinico‐pathologic patient data (Tumor size = 1 patient and pathologic stage = 3 patients) were excluded from the analysis. We presented the baseline characteristics of the TNBC patients including the treatments modalities they have received on (Table S2). The GSE119262 dataset was used to analyze mTOR inhibitor treatment response in patients with breast cancer. The list for the MTORC1 gene set was downloaded from the Molecular Signature Database (https://www.gsea‐msigdb.org/gsea/msigdb). The treatment profile of the TCGA‐BRCA patients was downloaded from the cBioPortal database (https://www.cbioportal.org/) and presented in Tables S2 and S6. DEGs were obtained using a cutoff value of log2FC ≥ 1 or ≤ 1, and *p* < 0.05. A Science and Research Plot (SR plot) was used to draw the heat maps.

### 2.2 Construction and Validation of a Risk Scoring Model Based on the DEGs in the MTORC1 Gene Set

Univariate and multivariate Cox regression analyses of the overall survival of TNBC patients were performed to identify prognosis‐related genes among the differentially expressed genes. The coefficient values used to establish the risk score were derived from the multivariate cox proportional hazard regression analyses. The risk score was calculated as follows: Risk Score = Coeff 1*gene expression 1 + Coeff 2*gene expression 2. The median risk score was used to divide the TNBC patients in TCGA dataset (*n* = 123) into high‐ and low‐risk groups. To assess the efficacy of the generated risk score, Kaplan–Meier overall survival (OS) and receiver operating characteristic (ROC) curves were plotted using the Kaplan–Meier plotter and SPSS, respectively.

### 2.3 MTORC1 Gene Set Scoring

Using the above mentioned list for MTORC1 gene set and the TCGA dataset which contains the normalized gene expression RNA‐Seq data, we estimated the MTORC1 gene set activity between the Normal mammary, Non‐TNBC, and TNBC samples. MTORC1 gene set activity was calculated using the sum of expressions of all the 200 genes in each sample. The list of MTORC1 gene set from the Molecular Signature Database is provided in Table S1. Spearman correlation coefficients were used to study the association between MTORC1 gene set activity and the IC50 values of Rapamycin and Temsirolimus.

### 2.4 Reagents

Rapamycin, Temsirolimus, and Everolimus were purchased from MedChemExpress LLC (NJ, USA).

### 2.5 Cell Culture

Human breast cancer cell lines HCC1937, HCC1806, HCC38, Hs578T, and MDA‐MB‐231 and human lung adenocarcinoma cell line A549 were purchased from the American Type Culture Collection (ATCC). HCC1937, HCC1806, HCC38, and A549 cells were cultured in RPMI‐1640 medium. Hs578T and MDA‐MB‐231 cells were cultured in Dulbecco's Modified Eagle's medium (DMEM) and Leibovitz's (L‐15) medium with 10% FBS at 37°C with and without 5% CO_2_, respectively. A549 cells and U‐20S lysate were used as positive control for FADS2 and TOMM40, respectively.

### 2.6 Western Blot Analysis

Total protein (20–100 μg) was extracted using RIPA lysis buffer (Cambridge, UK) and loaded for electrophoretic separation on SDS/polyacrylamide gels before transfer onto the PVDF membrane. Membranes were immersed in blocking buffer (TFU‐BLP500; TOOLS Biotech, New Taipei city, Taiwan) for 1–2 h, followed by incubation with primary antibodies diluted in blocking buffer, FADS2 Rabbit pAb (1:2000) (#A10270; ABclonal, Woburn, USA), TOMM40 Rabbit pAb (1:2000) (#A3213, ABclonal, Woburn, USA), and GAPDH (1:10000) (GTX100118; GeneTex, Irvine, USA) overnight. After extensive washing with TBST, the membranes were incubated with secondary antibody (1:10000), goat anti‐rabbit (C04003; Croyez, Taipei, Taiwan), diluted in blocking buffer for 1 h at room temperature, followed by extensive washing with TBST and visualization using the T‐Pro LumiDura Chemiluminescent Detection Kit (M) (T‐Pro Biotechnology, New Taipei, Taiwan). The original full‐length western blot gels are shown in Figure S1.

### 2.7 Immunohistochemistry (IHC) Staining

Tissue microarray sections purchased from SuperBioChips (Seoul, South Korea) were deparaffinized in xylene and washed with alcohol (100%, 95%, 70%, and 50%) and phosphate‐buffered saline (PBS). Antigen retrieval was performed using citrate buffer (pH 6.0) at 115°C for 15 min followed by cooling. Endogenous peroxidase activity was blocked with 3% hydrogen peroxide (PolyDetector Peroxidase Blocker, BSB 0050). The sections were incubated with blocking buffer (1:20 normal goat serum in PBS), followed by incubation with primary antibodies (TOMM40 #A3213, ABclonal, and FADS2 #A10270; ABclonal) at a dilution of 1:500 in PBS overnight at 4°C. After washing with PBS, the sections were treated with a secondary antibody (N‐Histofine Simple Stain Mouse MAX PO (R)) and developed using DAB (PolyDetector Liquid DAB HRP Brown Kit). Counterstaining was performed with hematoxylin. Slides were dehydrated, cleared in xylene, mounted, and observed under a microscope.

### 2.8 MTT Assay

HCC1937 and HS578T cells were seeded at a density, which was determined by their 70% growth rate at the end of the experiment, 5 × 10^4^/mL and 2 × 10^4^/mL, respectively, into a 96‐well culture plate and incubated at 37°C. After cultivation for 24 h, the cells were treated with mTOR inhibitors at the designated concentrations and incubated for another 96 h. At the end of the incubation, 10 μL of 3‐(4,5‐dimethylthiazol‐2‐yl)‐2,5‐diphenyltetrazoliumbromide (MTT) (Molecular Probe, Invitrogen, CA, USA) stock solution (5 mg/mL) was added to each well. The conversion of MTT to formazan by viable cells was performed at 37°C for 4 h. Subsequently, 150 μL DMSO solution was added to each well to solubilize the formazan precipitate. Formazan levels were determined by measuring optical density at 595 nm using an ELISA reader for calculating cell survival rates.

### 2.9 Statistical Analysis

MS Excel 2016 and GEO2R were used to generate DEGs and risk scoring. SPSS. V18 (Released 2009; SPSS Inc., Chicago, USA) was used for Cox regression analysis and ROC curves. IC_50_ data from three independent biological replicates for each treatment in both cell lines is presented as the mean ± SEM of the treated cells. We have used the following formula for IC_50_ calculation, and Graph prism (four parameter) was used to draw the dose–response curve.

$$\begin{array}{cc} \mathrm{Log}\;\left(\mathrm{IC}50\right)\; & =\left(50-\text{Y1}\right)\times \left(\mathrm{Log}\;\left(\text{X2}\right)–\mathrm{Log}\;\left(\text{X1}\right)\right)/\left(Y2-\text{Y1}\right)\\ & +\mathrm{Log}\;\left(\text{X2}\right) \end{array}$$

where X1 = lower concentration; X2 = higher concentration; Y1 = viability at X1; Y2 = viability at X2. Mann–Whitney *U* test was used for statistical significance at *p* < 0.05.

## 3 Results

### 3.1 FADS2 and TOMM40 Genes of the MTORC1 Gene Set Are Prognostic Factors for TNBC

**FIGURE 1:**
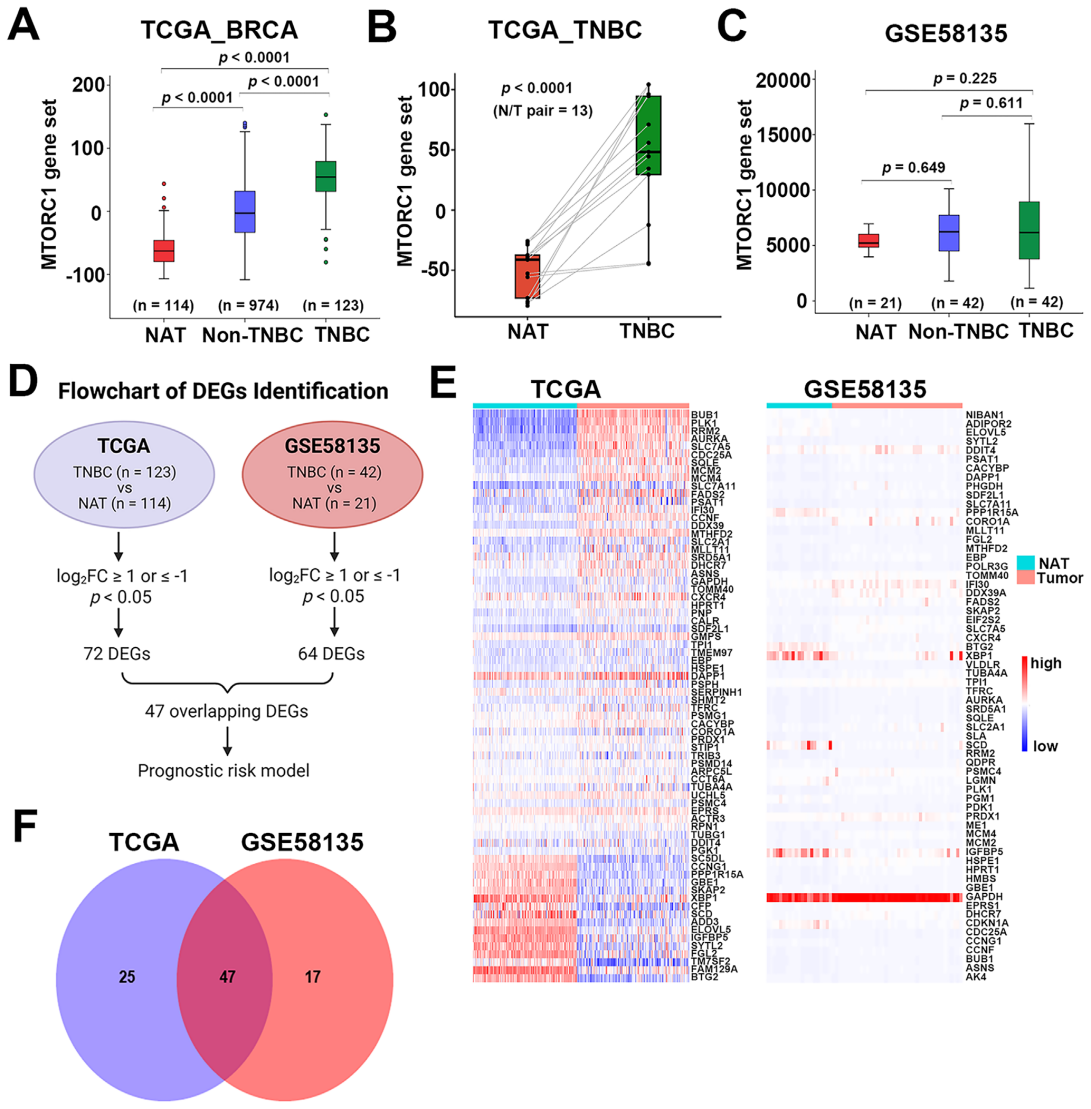
Upregulated and downregulated MTORC1 genes identified in TNBC tumors from the TCGA and GSE58135 datasets. (A–C) The transcription profile of the MTORC1 gene set in the normal adjacent tissue (NAT)/non‐TNBC/TNBC (A) and paired NAT/TNBC (B) from the TCGA database and in the NAT/non‐TNBC/TNBC from the GSE58135 dataset (C). (D) Flowchart for identifying the differentially expressed genes (DEGs) of the MTORC1 gene set between TNBC and NAT from the TCGA database and the GSE58135 dataset. (E) Heatmaps for the DEGs of the MTORC1 gene set in the NAT and TNBC from the TCGA database and the GSE58135 dataset. (F) Venn diagram for the overlapping DEGs of the MTORC1 gene set in the TCGA database and the GSE58135 dataset.

**FIGURE 2:**
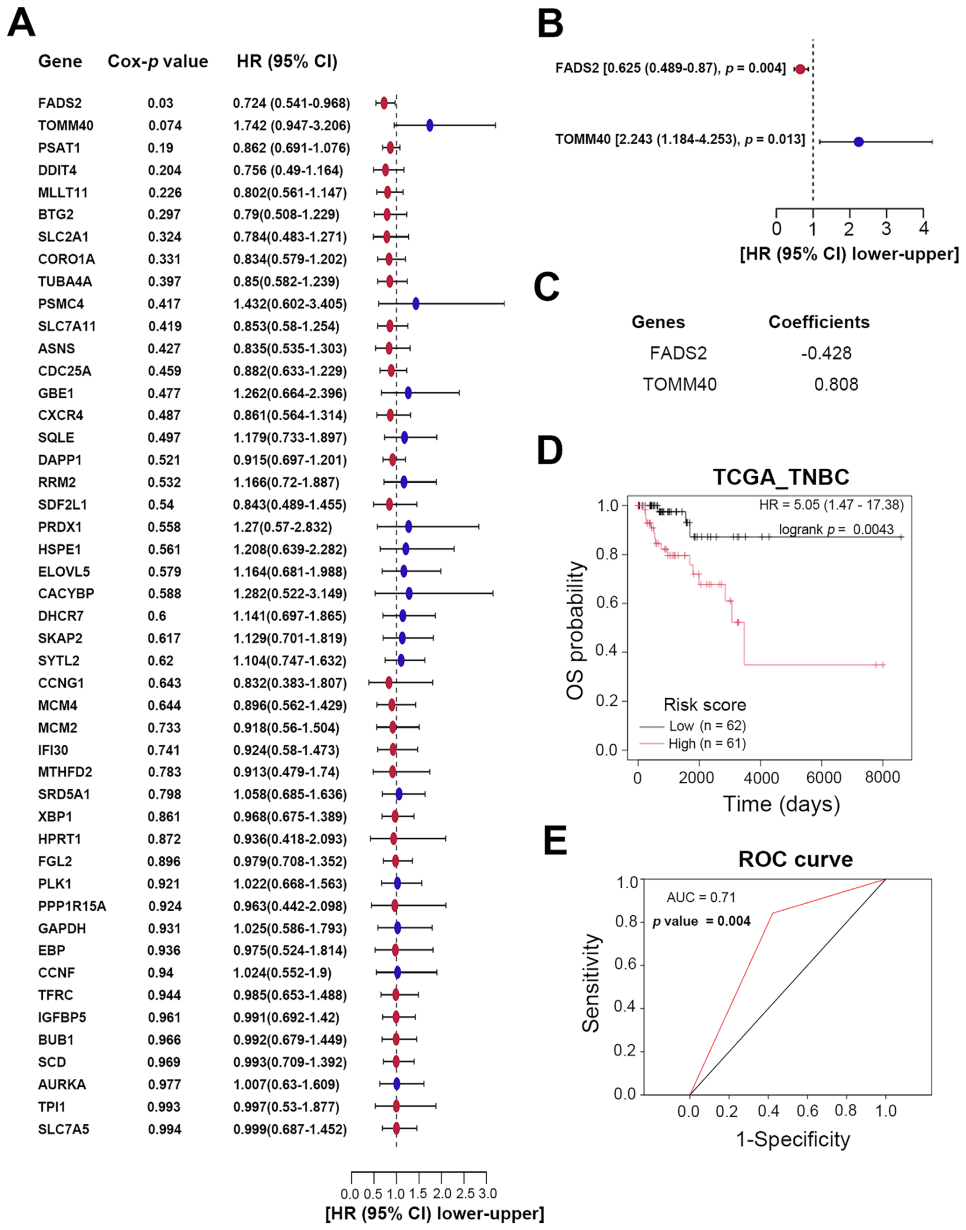
TOMM40 and FADS2 are independent prognostic biomarkers for TNBC. (A, B) Forest plots for cox regression analysis using univariate (A) and multivariate (B) modes for the 47 overlapping DEGs of the MTORC1 gene set and FADS2/TOMM40 genes, respectively, under the overall survival condition against TCGA TNBC patients. (C) Genes from the MTORC1 gene set selected to build a risk signature and the corresponding coefficients from the multivariate analysis. (D, E) The validation for the risk score of TOMM40/FADS2 genes using Kaplan–Meier analysis under overall survival (OS) condition (D) and receiver operating characteristic analysis (E) against TCGA TNBC patients. In C, patients were stratified by the median of the risk score.

We first investigated the transcriptional profile of the MTORC1 gene set (Table S1), which theoretically refers to mTORC1 activity in BRCA patients in TCGA and GSE58135 datasets. Our findings revealed that mTORC1 activity in TNBC tumor tissues was higher than that in non‐TNBC tumor and normal tissues of patients with TNBC (Figure 1A–C). We then analyzed differentially expressed genes (DEGs) in TNBC tumor tissues compared to normal adjusted tissues from TNBC patients from the TCGA database and GSE58135 dataset (Figure 1D). We found 72 and 64 genes included in the MTORC1 gene set in the DEGs of TCGA and GSE58135 TNBC tumor tissues, respectively, compared with normal TNBC tissues (Figure 1E). We further identified 47 overlapping genes between the DEGs of the MTORC1 gene set from TCGA and GSE58135 TNBC samples (Figure 1F). The baseline characteristics of TCGA‐TNBC patients used in the analysis are displayed on Table S2. Next, we performed Cox regression tests using univariate analysis under overall survival conditions for the 47 overlapping genes in the TCGA TNBC cohort. The data showed that FADS2 (HR = 0.724) was a protective factor, whereas TOMM40 (HR = 1.742) was a risk factor (Figure 2A). Using multivariate analysis, we found that FADS2 and TOMM40 were independent prognostic markers for predict favorable (HR = 0.652, *p* = 0.004) and poor (HR = 2.243, *p* = 0.013) overall survival rates, respectively, in TCGA TNBC patients (Figure 2B). We further established a prognostic risk‐scoring model using FADS2 and TOMM40 mRNA levels in primary tumors from TCGA TNBC patients and their coefficient values (Figure 2C) in multivariate analysis and validated its prognostic significance. Kaplan–Meier analysis demonstrated that a significantly higher risk score (*p* = 0.0043) predicts poor overall survival in TCGA TNBC patients (Figure 2D). Receiver operating characteristic (ROC) analysis also showed that the risk score of FADS2 and TOMM40 expression is a powerful biomarker to predict prognosis in TCGA TNBC cohort (Figure 2E). Cox regression analysis revealed that the generated risk score was an independent factor compared with other clinical confounders for TNBC survival Table 1.

**TABLE 1:** Cox regression test using univariate and multivariate analysis for the risk scoring signature of TOMM40 and FADS2 in TCGA TNBC patients.

Variable	Univariate			Multivariate		
	HR	95% CI	*p*	HR	95% CI	*p*
Age						
< 62	Reference					
≥ 62	1.622	0.609–4.322	0.333	1.062	0.358–3.151	0.914
pT						
T1–T2	Reference					
T3–T4	3.121	1.117–8.722	**0.03**	0.635	0.162–2.499	0.516
pN						
No	Reference					
Yes	3.63	1.377–9.568	**0.009**	1.616	0.409–6.389	0.494
Stage						
I–II	Reference					
III–IV	5.906	2.373–14.7	**< 0.0001**	5.714	1.202–27.152	**0.028**
Risk score						
Low	Reference					
High	5.236	1.519–18.057	**0.009**	5.062	1.401–18.294	**0.013**

*Note:* Cox regression test using univariate and multivariate analyses for the risk scoring signature of TOMM40 and FADS2 genes against TCGA TNBC patients under the overall survival condition. Significant differences (*p* < 0.05) are indicated in bold.

### 3.2 The Ratio of TOMM40/FADS2 Gene Expression Refers to a Poorer Clinical Outcome in TNBC Patients

**FIGURE 3:**
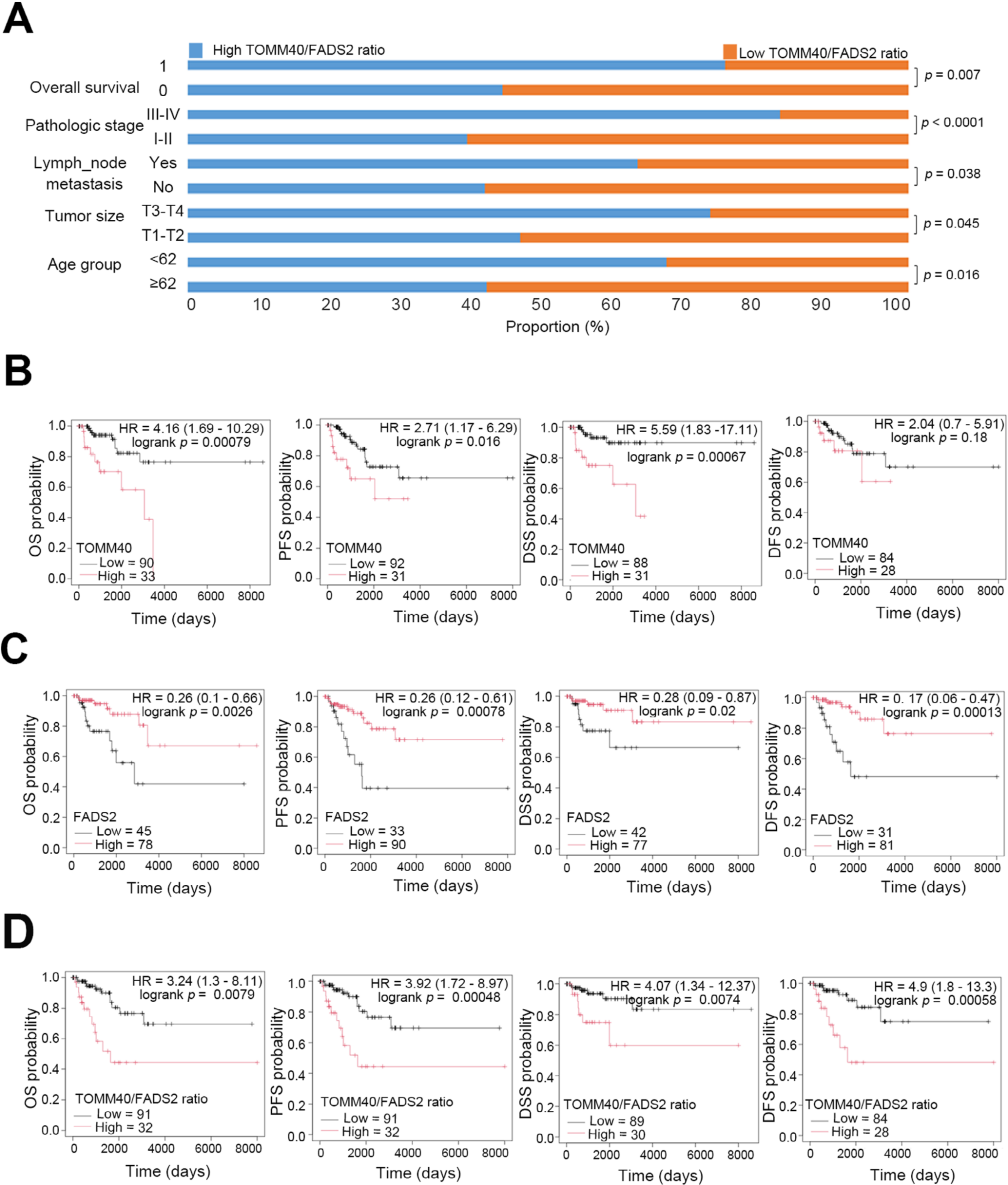
A High TOMM40/FADS2 expression ratio refers to a poor prognosis in TNBC. (A) The histogram for the TOMM40/FADS2 ratio associations with the clinicopathologic characteristics of TNBC patients. (B–D) Kaplan–Meier analyses for the TOMM40 (B), FADS2 (C), and TOMM40/FADS2 expression ratio (D) using overall survival (OS), progression‐free index (PFI), disease‐specific survival (DSS), and disease‐free survival (DFS) conditions under a minimized *p* values against TCGA TNBC patients.

To ascertain whether the TOMM40/FADS2 expression ratio could also be a prognostic biomarker for TNBC, we analyzed the association of the TOMM40, FADS2 and TOMM40/FADS2 ratio with clinicopathological data in TCGA TNBC cohort. The data showed that TOMM40/FADS2 ratio has a superior clinical correlation compared to either TOMM40 or FADS2 alone (Table S3) where a higher gene expression ratio of TOMM40/FADS2 was significantly (*p* < 0.05) correlated with old age (≥ 62 years), advanced T stage, lymph node metastasis, advanced pathologic stage, and poor overall survival outcome (Figure 3A). Although the protein expression levels of TOMM40 and FADS2 in TCGA TNBC dataset are incomplete (Table S4), however the immunohistochemistry (IHC) staining also revealed inverse protein expression of TOMM40 and FADS2 in the breast cancer tissues (Figure S2A,B). The clinico‐pathologic characteristics of breast cancer patients in tissue microarray slide are displayed on (Table S5). Furthermore, Kaplan–Meier analyses demonstrated that although TOMM40 and FADS2 have a significant correlation in TNBC prognosis (Figure 3B,C), a higher gene expression ratio of TOMM40/FADS2 has a superior association with poor overall, progression‐free, disease‐specific, and disease‐free survival rates in TCGA TNBC cohort (Figure 3D). Similar trends were observed in Kaplan–Meier analysis of TNBC in GSE202203‐GPL11154, GSE21653, GSE2603 and GSE65194 datasets (Figure S3A–D). Cox regression analysis using univariate and multivariate models indicated that the gene expression ratio of TOMM40/FADS2 is a prognostic factor for TNBC Table 2. We further found that high TOMM40/FADS2 expression ratio could predict a worse survival in TNBC patients receiving chemotherapy‐based adjuvant therapy compared to TNBC patients who received non‐chemotherapy‐based adjuvant therapy (Figure S4A–C). The treatment modalities received by each TNBC patient in TCGA‐BRCA dataset downloaded from cBioPortal database are displayed on (Table S6).

**TABLE 2:** Cox regression test using univariate and multivariate analysis for the gene expression ratio of TOMM40 and FADS2 in TCGA TNBC patients.

Variable	Univariate			Multivariate		
	HR	95% CI	*p*	HR	95% CI	*p*
Age						
< 62	Reference					
≥ 62	1.622	0.609–4.322	0.333	1.278	0.436–3.748	0.655
pT						
T1–T2	Reference					
T3–T4	3.121	1.117–8.722	**0.03**	0.619	0.161–2.381	0.485
pN						
No	Reference					
Yes	3.63	1.377–9.568	**0.009**	1.72	0.449–6.592	0.429
Stage						
I–II	Reference					
III–IV	5.906	2.373–14.7	**< 0.0001**	4.126	0.865–19.672	0.075
TOMM40/FADS2						
Low	Reference					
High	3.591	1.435–8.988	**0.006**	2.387	0.833–6.837	0.105

*Note:* Cox regression analysis using univariate and multivariate modes for TOMM40/FADS2 expression ratio and clinicopathological characteristics of TCGA TNBC patients under overall survival conditions. Significant differences (*p* < 0.05) are indicated in bold.

### 3.3 TOMM40/FADS2 Gene Expression Ratio Predicts the Therapeutic Efficacy of mTOR Inhibitors in TNBC

**FIGURE 4:**
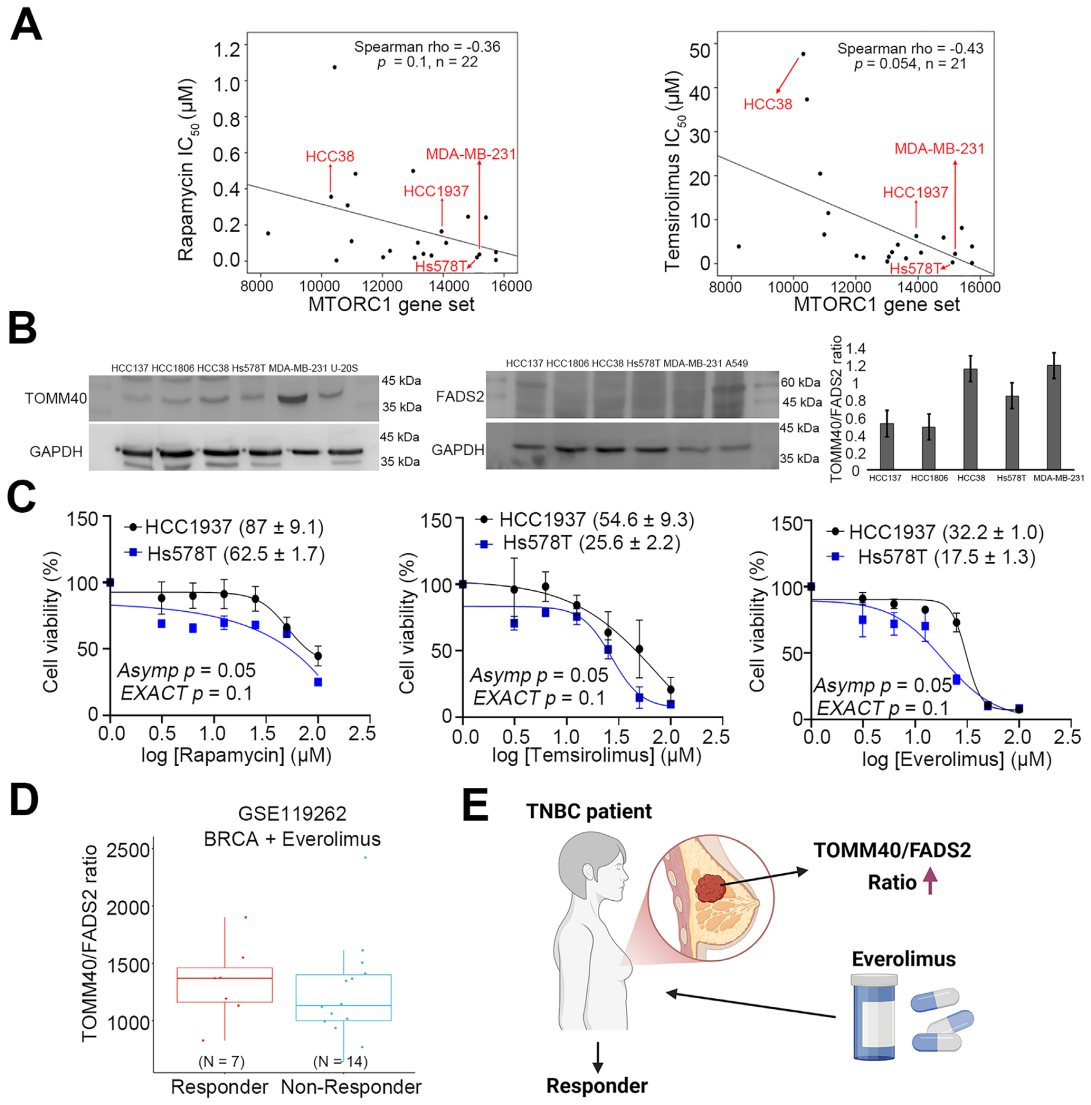
TNBC cells with a high TOMM40/FADS2 expression ratio are more sensitive to mTOR inhibitors treatment. (A) The Scatchard plots for the gene expression levels of the MTORC1 gene set and the IC50 concentrations of mTOR inhibitors Rapamycin (left) and Temsirolimus (right) from the Genomics of Drug Sensitivity in Cancer database in the tested TNBC cell lines. Spearman correlation coefficient analysis was used to assess the correlation between MTORC1 gene set activity and IC50 values of rapamycin and temsirolimus. (B) Western blot analysis for the protein levels of TOMM40/FADS2 (upper) and the histogram for the TOMM40/FADS2 expression ratio (lower) in TNBC cell lines HCC1937, HHC1806, HCC38, Hs578T and MDA‐MB‐231. A549 and U‐20S were used as positive controls for FADS2 and TOMM40 respectively and GAPDH was used as an internal control of protein loading. (C) Cell viability of HCC1937 and Hs578T cells after mTOR inhibitors treatment, Rapamycin (left), Temsirolimus (middle) and Everolimus (right). The IC50 concentrations of these mTOR inhibitors are presented in mean ± SEM as shown in the inserts. (D) The box plot for the TOMM40/FADS2 expression ratio in the preoperative primary tumors of breast cancer patients who were responders or nonresponders to the neoadjuvant treatment with Everolimus. (E) The proposed model for the clinical application of TOMM40/FADS2 expression ratio predicts the therapeutic effectiveness of mTOR inhibitors, for example, Everolimus, on TNBC patients.

We found that the transcriptional profile of the MTORC1 gene set, derived from the Cancer Cell Line Encyclopedia database on the Xena website (https://xena.ucsc.edu/welcome‐to‐ucsc‐xena/), was negatively correlated with the half‐inhibitory concentrations (IC_50_) of mTOR inhibitors Rapamycin and Temsirolimus, obtained from the website (https://www.cancerrxgene.org/) of Genomics of Drug Sensitivity in Cancer, in the tested 22 and 21 TNBC cell lines, respectively (Figure 4A). The list of TNBC cells used for the analysis and their IC_50_ values to mTOR inhibitors treatment are displayed on (Table S7). Intriguingly, we found that four TNBC cell lines, HCC1937, HCC38, Hs578T and MDA‐MB‐231 which exhibit a poorer and stronger metastatic capacity [9], displayed a different feature in the transcriptional profile of the MTORC1 gene set and the IC_50_ of Rapamycin and Temsirolimus (Figure 4A). To further determine whether the TOMM40/FADS2 gene expression ratio could be used as a biomarker for predicting the anticancer effectiveness of mTOR inhibitors in TNBC, we measured the protein expression levels of TOMM40 and FADS2 in HCC1937, HCC1806, HCC38, Hs578T and MDA‐MB‐231 cells. Western blot analysis revealed that the TOMM40/FADS2 protein expression ratio in highly metastatic Hs578T and MDA‐MB‐231 cells was higher than that in poorly metastatic HCC1937 cells (Figure 4B). Importantly, the cell‐based cytotoxicity assay revealed that the highly metastatic Hs578T cells, compared to the poorly metastatic HCC1937 cells, were more sensitive to treatment with three mTOR inhibitors: Rapamycin, Temsirolimus and Everolimus (Figure 4C). The cell viabilities of HCC1937 and Hs578T cells at six different concentrations of the three mTOR inhibitors used are presented in Table S8. In addition, by re‐analyzing the transcriptional profile of the GSE119262 dataset, we found that a higher gene expression ratio of TOMM40/FADS2 existed in the preoperative breast cancer tissues derived from the responders, which was determined by the reduced phosphorylated Akt protein level in the postoperative breast cancer tissues after everolimus therapy (Figure 4D).

## 4 Discussion

Two hundred genes of HALLMARK_MTORC1_SIGNALING from the Molecular Signatures Database were used to construct a gene signature of mTORC1 pathway‐related genes (mTRGs) in malignant rhabdoid tumors of the kidney (MRTK), an infrequent childhood malignant tumor. A higher expression level of mTRGs is associated with a poor overall survival rate in patients with MRTK [10]. In addition, these mTORC1‐related genes were employed to generate a risk score using univariate and multivariate Cox regression analyses in patients with osteosarcoma. High‐risk patients have a significantly poor prognosis, lower immune infiltration levels of multiple immune cells, and are prone to cancer metastasis [11]. Here, we found that TOMM40 and FADS2 from the HALLMARK_MTORC1_SIGNALING gene set were prognostic genes for poor and favorable prognosis, respectively, and that their related risk score could be independent prognostic biomarkers for predicting overall survival in TNBC patients. Moreover, their expression ratio, as well as the transcription profile of the MTORC1 gene set, could potentially serve as predictive biomarkers for the anticancer effectiveness of mTOR inhibitors in TNBC. Although the clinical application of the TOMM40/FADS2 expression ratio in predicting anticancer efficacy requires further validation, our data provide a potential biomarker for TNBC sensitivity to mTOR inhibitor treatment (Figure 4E).

TOMM40 is a key element for the translocase of the mitochondrial outer membrane (TOM), which is crucial for transporting proteins into the mitochondria and is a mitochondrial autophagy‐related gene [12]. TOMM40 upregulation in primary tumors compared to that in adjacent normal tissues has been reported in uveal melanoma, lung adenocarcinoma, colorectal, and breast cancers [13, 14, 15, 16]. TOMM40 upregulation has been linked to its oncogenic role in these cancers. In the TNBC cell line MDA‐MB231, TOMM40 knockdown inhibits cellular proliferation, migration, and invasiveness [16]. Here, we found that TOMM40 expression in highly metastatic Hs578T cells was higher than that in poorly metastatic HCC1937 cells. Although the individual expression of TOMM40 was insignificant, the TOMM40/FADS2 expression ratio was significantly correlated with advanced T stage and lymph node metastasis in TNBC patients. These findings suggest that TOMM40 may be a potential target for treating metastatic TNBC in future clinical trials. However, further experiments are needed to explore the mechanism of transcriptional regulation of the TOMM40 gene by the mTORC1 pathway, and the involvement of TOMM40 in mTORC1‐restrained autophagy formation.

FADS2 (D‐6‐fatty acid desaturase) is a rate‐limiting enzyme in the synthesis of mammalian polyunsaturated fatty acids (PUFAs) and belongs to mitochondrial metabolism‐related genes [17]. PUFAs are important structural determinants [18]. FADS2 upregulation has been reported in bladder cancer, cholangiocarcinoma, lung squamous cell carcinoma, colorectal cancer, and breast cancer in comparison with their respective normal tissues [17, 19, 20, 21, 22], and its upregulation correlates with poor prognosis, which conflicts with our current findings in TNBC. Moreover, a previous report demonstrated that FADS2 knockdown in the TNBC cell line HCC1806 suppressed cellular proliferation, migration, and invasion [17]. Therefore, further studies are needed to confirm the functional roles of FADS2 in TNBC progression and its connection with mTORC1 and TOMM40 pathways.

Previous studies have reported a role of FADS2 in apoptotic sensitivity [23], temozolomide/irradiation‐induced cell death in GBM tumors [24], and sensitivity/resistance of cancer cells to SCD1 inhibitors [25]. In addition, FADS2 was reported to mediate resistance to the PI3K/mTOR inhibitor BEZ235 in NSCLC [26]. In TNBC, FADS2 and SCD1 regulation modulates ferroptosis sensitivity through PI3K/Akt and MAPK signaling [27]. Here, we report that a lower TOMM40/FADS2 ratio predicts poor responsiveness to mTOR inhibitors in cell‐based cytotoxic assays and in patients with breast cancer receiving Everolimus monotherapy. However, the comprehensive mechanism by which FADS2 upregulation leads to resistance to mTOR inhibitors, possibly by inducing intracellular ferroptosis, requires further investigation in TNBC patients.

## 5 Conclusion

Here, we present a new therapeutic strategy using the TOMM40/FADS2 expression ratio to predict the cellular sensitivity to mTOR inhibitor treatment in TNBC. Our findings are of great importance in precision cancer medicine using the TOMM40/FADS2 expression ratio to discriminate patients with TNBC who would benefit from mTOR‐targeted therapy.

## Author Contributions

**Salman Mohamed Farah:** conceptualization (lead), formal analysis (lead), investigation (lead), methodology (equal), writing – original draft (lead). **Jing‐Quan Zheng:** formal analysis (supporting), investigation (supporting). **Leon Tsung‐Ju Lee:** formal analysis (supporting), investigation (supporting). **Hsiao‐Wei Lu:** methodology (supporting), resources (supporting). **Wen‐Ke Wang:** methodology (supporting), resources (supporting). **Chia‐Hao Kuei:** methodology (supporting), resources (supporting). **Hui‐Yu Lin:** conceptualization (supporting), funding acquisition (equal), methodology (equal), resources (equal), writing – review and editing (equal). **Hui‐Wen Chiu:** conceptualization (supporting), formal analysis (supporting), funding acquisition (equal), investigation (supporting), methodology (equal), resources (equal), writing – review and editing (equal). **Yuan‐Feng Lin:** conceptualization (lead), formal analysis (lead), funding acquisition (equal), investigation (lead), methodology (equal), resources (equal), writing – original draft (lead), writing – review and editing (equal).

## Funding

This study was supported by the Cardinal Tien Hospital (CTH114A‐2003 to Hui‐Yu Lin) and the National Science and Technology Council of Taiwan (NSTC 114‐2314‐B‐038‐050‐MY3 to Hui‐Wen Chiu and NSTC 114‐2320‐B‐038‐041‐MY3 to Yuan‐Feng Lin).

## Ethics Statement

The authors have nothing to report.

## Consent

The authors have nothing to report.

## Conflicts of Interest

The authors declare no conflicts of interest.

## Supporting information


**Table S1:** List of 200 MTORC1‐related genes downloaded from the molecular signature database.
**Table S2:** Baseline characteristics of TNBC patients in TCGA dataset.
**Table S3:** Associations of TOMM40 and FADS2 expressions and the clinicopathologic characteristics of TNBC patients in TCGA dataset.
**Table S4:** TOMM40 and FADS2 protein expression levels in TCGA TNBC patients.
**Table S5:** Clinico‐pathologic characteristics of breast cancer patients used for IHC analysis.
**Table S6:** Treatment profile of TCGA‐TNBC patients.
**Table S7:** List of TNBC cell lines from Genomics of Drug Sensitivity database used for the analysis of mTOR inhibitor treatment response.
**Table S8:** Cell viabilities of mTOR inhibitors treated TNBC cells.
**Figure S1:** Originally uncut blots from Western blot analyses for TOMM40 (A) and FADS2 (B) with their respective GAPDH (Right) in TNBC cell lines HCC1937, HCC1806, HCC38, Hs578T, MDA‐MB‐231 and positive controls A549 and U‐20S.
**Figure S2:** The representatives of high (A) and low (B) TOMM40/FADS2 ratios in IHC staining against breast cancer tissues.
**Figure S3:** (A–D) Kaplan–Meier analyses for the TOMM40/FADS2 expression ratio using overall survival (OS) in GSE2022203‐GPL11154 (A) and recurrence‐free survival (RFS) in GSE21653 (B) GSE2603 (C) and GSE65194 (D) conditions under a minimized *p* values against GEO TNBC patients.
**Figure S4:** (A–C) Kaplan–Meier analyses for the TOMM40 (A), FADS2 (B) and TOMM40/FADS2 expression ratio (C) in non‐chemotherapy based (upper) and chemotherapy based treatment (lower) using overall survival (OS), progression‐free index (PFI), disease‐specific survival (DSS) and disease‐free survival (DFS) conditions under a minimized *p* values against TCGA TNBC patients.

## Data Availability

The datasets analyzed in this study were obtained from a public database. The data were downloaded from https://tcga.xenahubs.net, https://www.ncbi.nlm.nih.gov/geo/, and www.cancerRxgene.org.
