# Supplementary material for: TOMM40/FADS2 Expression Ratio Predicts the Sensitivity to mTOR Inhibitors in Triple‐Negative Breast Cancer
Source: Cancer Med. 2026 Jul 18;15(7):e72089. doi: 10.1002/cam4.72089 (PMC13380028; doi:10.1002/cam4.72089)
Supplement: Supplementary file 1 — Table S1: List of 200 MTORC1‐related genes downloaded from the molecular signature database. Table S2: Baseline characteristics of TNBC patients in TCGA dataset. Table S3: Associations of TOMM40 and FADS2 expressions and the clinicopathologic characteristics of TNBC patients in TCGA dataset. Table S4: TOMM40 and FADS2 protein expression levels in TCGA TNBC patients. Table S5: Clinico‐pathologic characteristics of breast cancer patients used for IHC analysis. Table S6: Treatment profile of TCGA‐TNBC patients. Table S7: List of TNBC cell lines from Genomics of Drug Sensitivity database used for the analysis of mTOR inhibitor treatment response. Table S8: Cell viabilities of mTOR inhibitors treated TNBC cells. Figure S1: Originally uncut blots from Western blot analyses for TOMM40 (A) and FADS2 (B) with their respective GAPDH (Right) in TNBC cell lines HCC1937, HCC1806, HCC38, Hs578T, MDA‐MB‐231 and positive controls A549 and U‐20S. Figure S2: The representatives of high (A) and low (B) TOMM40/FADS2 ratios in IHC staining against breast cancer tissues. Figure S3: (A–D) Kaplan–Meier analyses for the TOMM40/FADS2 expression ratio using overall survival (OS) in GSE2022203‐GPL11154 (A) and recurrence‐free survival (RFS) in GSE21653 (B) GSE2603 (C) and GSE65194 (D) conditions under a minimized p values against GEO TNBC patients. Figure S4: (A–C) Kaplan–Meier analyses for the TOMM40 (A), FADS2 (B) and TOMM40/FADS2 expression ratio (C) in non‐chemotherapy based (upper) and chemotherapy based treatment (lower) using overall survival (OS), progression‐free index (PFI), disease‐specific survival (DSS) and disease‐free survival (DFS) conditions under a minimized p values against TCGA TNBC patients. [file CAM4-15-e72089-s001.docx]

**SUPPLEMENTARY TABLE 1.** List of 200 MTORC1-related genes downloaded from the Molecular Signature Database

| **No.** | **Gene** | **No.** | **Gene** | **No.** | **Gene** | **No.** | **Gene** | **No.** | **Gene** |
| --- | --- | --- | --- | --- | --- | --- | --- | --- | --- |
| 1  2  3  4  5  6  7  8  9  10  11  12  13  14  15  16  17  18  19  20  21  22  23  24  25  26  27  28  29  30  31  32  33  34  35  36  37  38  39  40 | ABCF2  ACACA  ACLY  ACSL3  ACTR2  ACTR3  ADD3  ADIPOR2  AK3L1  ALDOA  ARPC5L  ASNS  ATP2A2  ATP5G1  ATP6V1D  AURKA  BCAT1  BHLHE40  BTG2  BUB1  CACYBP  CALR  CANX  CCNF  CCNG1  CCT6A  CD9  CDC25A  CDKN1A  CFP  COPS5  CORO1A  CTH  CTSC  CXCR4  CYB5B  CYP51A1  DAPP1  DDIT3  DDIT4 | 41  42  43  44  45  46  47  48  49  50  51  52  53  54  55  56  57  58  59  60  61  62  63  64  65  66  67  68  69  70  71  72  73  74  75  76  77  78  79  80 | DDX39  DHCR24  DHCR7  DHFR  EBP  EDEM1  EEF1E1  EGLN3  EIF2S2  ELOVL5  ELOVL6  ENO1  EPRS  ERO1L  ETF1  FADS1  FADS2  FDXR  FGL2  FKBP2  G6PD  GAPDH  GBE1  GCLC  GGA2  GLA  GLRX  GMPS  GOT1  GPI  GSK3B  GSR  GTF2H1  HK2  HMBS  HMGCR  HMGCS1  HPRT1  HSP90B1  HSPA4 | 81  82  83  84  85  86  87  88  89  90  91  92  93  94  95  96  97  98  99  100  101  102  103  104  105  106  107  108  109  110  111  112  113  114  115  116  117  118  119  120 | HSPA5  HSPA9  HSPD1  HSPE1  IDH1  IDI1  IFI30  IFRD1  IGFBP5  IMMT  INSIG1  ITGB2  LDHA  LDLR  LGMN  LTA4H  M6PR  MAP2K3  MCM2  MCM4  ME1  MLLT11  MTHFD2  MTHFD2L  NAMPT  NFIL3  NFKBIB  NFYC  FAM129A  NMT1  NUFIP1  NUP205  NUPR1  P4HA1  PDAP1  PDK1  PFKL  PGK1  PGM1  PHGDH | 121  122  123  124  125  126  127  128  129  130  131  132  133  134  135  136  137  138  139  140  141  142  143  144  145  146  147  148  149  150  151  152  153  154  155  156  157  158  159  160 | PIK3R3  PITPNB  PLK1  PLOD2  PNO1  PNP  POLR3G  PPA1  PPIA  PPP1R15A  PRDX1  PSAT1  PSMA3  PSMA4  PSMB5  PSMC2  PSMC4  PSMC6  PSMD12  PSMD13  PSMD14  PSME3  PSMG1  PSPH  QDPR  RAB1A  RDH11  RIT1  RPA1  RPN1  RRM2  RRP9  SC5DL  SCD  SDF2L1  SEC11A  SERP1  SERPINH1  SHMT2  SKAP2 | 161  162  163  164  165  166  167  168  169  170  171  172  173  174  175  176  177  178  179  180  181  182  183  184  185  186  187  188  189  190  191  192  193  194  195  196  197  198  199  200 | SLA  SLC1A4  SLC1A5  SLC2A1  SLC2A3  SLC37A4  SLC6A6  SLC7A11  SLC7A5  SLC9A3R1  SORD  SQLE  SQSTM1  SRD5A1  SSR1  STARD4  STC1  STIP1  SYTL2  TBK1  TCEA1  TES  TFRC  TM7SF2  TMEM97  TOMM40  TPI1  TRIB3  TUBA4A  TUBG1  TXNRD1  UBE2D3  UCHL5  UFM1  UNG  USO1  VLDLR  WARS  XBP1  YKT6 |

**SUPPLEMENTARY TABLE 2.** Baseline characteristics of TNBC patients in TCGA dataset

| **Variable** | **Frequency (n= 123)** | **Percentage (%)** |
| --- | --- | --- |
| **Age group** | | |
| <62  ≥62 | 88  35 | 72  28 |
| **Gender** | | |
| Female  Male | 123  0 | 100  0 |
| **Menopause status** | | |
| Pre  Post  Indeterminate/NA | 39  69  15 | 32  56  12 |
| **Tumor size** | | |
| T1-T2  T3-T4  Indeterminate | 108  14  1 | 88  11  1 |
| **Lymph node metastasis** | | |
| No  Yes | 78  45 | 63  37 |
| **Pathologic stage** | | |
| I-II  III-IV  Indeterminate | 98  22  3 | 80  18  2 |
| **OS** | | |
| Live  dead | 104  19 | 85  15 |
| **Progression free index** | | |
| Live  dead | 100  23 | 81  19 |
| **Disease specific survival** | | |
| Live  Dead  NA | 106  13  4 | 86  11  3 |
| **Disease free survival** | | |
| Live  Dead  NA | 96  16  11 | 78  13  9 |
| **First line therapy** | | |
| Surgery | 123 | 100 |
| **Adjuvant therapy** | | |
| Chemotherapy based | 82 | 66.6 |
| Non-chemotherapy based | 41 | 33.4 |

Frequency table for the baseline characteristics of TCGA TNBC patients.

**SUPPLEMENTARY TABLE 3.** Associations of TOMM40 and FADS2 expressions and the clinico-pathologic characteristics of TNBC patients in TCGA dataset

| **Variable** | **TOMM40** | | | **FADS2** | | | **TOMM40/FADS2 ratio** | | |
| --- | --- | --- | --- | --- | --- | --- | --- | --- | --- |
|  | **High** | **Low** | **P value** | **High** | **Low** | **P value** | **High** | **Low** | **P value** |
| **Age group** | | | | | | | | | |
| <62  ≥62 | 21  11 | 64  24 | 0.449086 | 58  17 | 27  18 | **0.043135** | 18  15 | 67  20 | **0.015622** |
| **Tumor size** | | | | | | | | | |
| T1-T2  T3-T4 | 27  5 | 79  9 | 0.415348 | 70  5 | 36  9 | **0.027618** | 26  7 | 80  7 | **0.044847** |
| **Lymph node metastasis** | | | | | | | | | |
| No  Yes | 21  11 | 55  33 | 0.753414 | 52  23 | 24  21 | 0.078269 | 16  17 | 60  27 | **0.037633** |
| **Pathologic stage** | | | | | | | | | |
| I-II  III-IV | 24  8 | 74  14 | 0.255068 | 68  7 | 30  15 | **0.001004** | 19  14 | 79  8 | **2.66E-05** |
| **Overall survival** | | | | | | | | | |
| Live  dead | 22  10 | 79  9 | **0.005276** | 68  7 | 33  12 | **0.011799** | 23  10 | 78  9 | **0.007491** |
| **Total** | 32 | 88 |  | 75 | 45 |  | 33 | 87 |  |

Associations of TOMM40 and FADS2 expressions and the clinico-pathologic characteristics of TNBC patients in TCGA dataset using chi-square test of independence under overall survival conditions. Significant differences (*p* < 0.05) are indicated in bold.

**SUPPLEMENTARY TABLE 4.** TOMM40 and FADS2 protein expression levels in TCGA TNBC patients

| SAMPLE_ID | RPPA |  | CPTAC |  |
| --- | --- | --- | --- | --- |
|  | **TOMM40** | **FADS2** | **TOMM40** | **FADS2** |
| TCGA-A1-A0SK | NA | NA | NA | NA |
| TCGA-A1-A0SO | NA | NA | NA | NA |
| TCGA-A1-A0SP | NA | NA | NA | NA |
| TCGA-A2-A04P | NA | NA | NA | NA |
| TCGA-A2-A04Q | NA | NA | NA | NA |
| TCGA-A2-A04T | NA | NA | NA | NA |
| TCGA-A2-A04U | NA | NA | NA | NA |
| TCGA-A2-A0CM | NA | NA | 0.074 | -0.6164 |
| TCGA-A2-A0D0 | NA | NA | 1.8554 | 1.0726 |
| TCGA-A2-A0D2 | NA | NA | 0.2002 | 0.5464 |
| TCGA-A2-A0ST | NA | NA | NA | NA |
| TCGA-A2-A0SX | NA | NA | -0.0144 | -0.7984 |
| TCGA-A2-A0T0 | NA | NA | NA | NA |
| TCGA-A2-A0T2 | NA | NA | 0.1179 | -0.1776 |
| TCGA-A2-A0YE | NA | NA | NA | NA |
| TCGA-A2-A0YM | NA | NA | -0.5974 | -0.5839 |
| TCGA-A2-A1G6 | NA | NA | NA | NA |
| TCGA-A7-A0CE | NA | NA | 0.0154 | -1.4863 |
| TCGA-A7-A0DA | NA | NA | NA | NA |
| TCGA-A7-A26F | NA | NA | NA | NA |
| TCGA-A7-A26G | NA | NA | NA | NA |
| TCGA-A7-A26I | NA | NA | NA | NA |
| TCGA-A8-A07C | NA | NA | NA | NA |
| TCGA-A8-A07O | NA | NA | NA | NA |
| TCGA-A8-A08R | NA | NA | NA | NA |
| TCGA-A8-A09X | NA | NA | NA | NA |
| TCGA-AN-A04D | NA | NA | NA | NA |
| TCGA-AN-A0AL | NA | NA | 0.4544 | 0.9269 |
| TCGA-AN-A0AR | NA | NA | NA | NA |
| TCGA-AN-A0AT | NA | NA | NA | NA |
| TCGA-AN-A0FL | NA | NA | 1.1379 | 0.3957 |
| TCGA-AN-A0FX | NA | NA | NA | NA |
| TCGA-AN-A0G0 | NA | NA | NA | NA |
| TCGA-AN-A0XU | NA | NA | NA | NA |
| TCGA-AO-A03U | NA | NA | NA | NA |
| TCGA-AO-A0J2 | NA | NA | NA | NA |
| TCGA-AO-A0J4 | NA | NA | NA | NA |
| TCGA-AO-A0J6 | NA | NA | -0.2948 | -1.1815 |
| TCGA-AO-A0JL | NA | NA | 0.2448 | 0.2335 |
| TCGA-AO-A124 | NA | NA | NA | NA |
| TCGA-AO-A128 | NA | NA | NA | NA |
| TCGA-AO-A129 | NA | NA | NA | NA |
| TCGA-AO-A12F | NA | NA | -0.4258 | 1.4743 |
| TCGA-AQ-A04J | NA | NA | NA | NA |
| TCGA-AR-A0TS | NA | NA | NA | NA |
| TCGA-AR-A0TU | NA | NA | NA | NA |
| TCGA-AR-A0U0 | NA | NA | NA | NA |
| TCGA-AR-A0U1 | NA | NA | NA | NA |
| TCGA-AR-A0U4 | NA | NA | 0.3067 | -0.2925 |
| TCGA-AR-A1AI | NA | NA | NA | NA |
| TCGA-AR-A1AQ | NA | NA | 1.442 | 0.1552 |
| TCGA-AR-A1AR | NA | NA | NA | NA |
| TCGA-AR-A1AY | NA | NA | NA | NA |
| TCGA-AR-A256 | NA | NA | NA | NA |
| TCGA-B6-A0IE | NA | NA | NA | NA |
| TCGA-B6-A0IK | NA | NA | NA | NA |
| TCGA-B6-A0IQ | NA | NA | NA | NA |
| TCGA-B6-A0RE | NA | NA | NA | NA |
| TCGA-B6-A0RG | NA | NA | NA | NA |
| TCGA-B6-A0RN | NA | NA | NA | NA |
| TCGA-B6-A0RS | NA | NA | NA | NA |
| TCGA-B6-A0RT | NA | NA | NA | NA |
| TCGA-B6-A0RU | NA | NA | NA | NA |
| TCGA-B6-A0WX | NA | NA | NA | NA |
| TCGA-BH-A0AV | NA | NA | 0.0539 | -0.544 |
| TCGA-BH-A0B3 | NA | NA | NA | NA |
| TCGA-BH-A0B9 | NA | NA | NA | NA |
| TCGA-BH-A0BG | NA | NA | NA | NA |
| TCGA-BH-A0BL | NA | NA | NA | NA |
| TCGA-BH-A0BW | NA | NA | NA | NA |
| TCGA-BH-A0E0 | NA | NA | 1.1397 | -0.2205 |
| TCGA-BH-A0E6 | NA | NA | NA | NA |
| TCGA-BH-A0RX | NA | NA | NA | NA |
| TCGA-BH-A0WA | NA | NA | NA | NA |
| TCGA-BH-A18G | NA | NA | NA | NA |
| TCGA-BH-A18Q | NA | NA | 0.0545 | -1.072 |
| TCGA-BH-A18T | NA | NA | NA | NA |
| TCGA-BH-A18V | NA | NA | 0.9098 | 1.3619 |
| TCGA-BH-A1EW | NA | NA | NA | NA |
| TCGA-BH-A1FC | NA | NA | NA | NA |
| TCGA-C8-A12V | NA | NA | 0.1865 | -0.0864 |
| TCGA-C8-A131 | NA | NA | 0.0434 | 0.6967 |
| TCGA-C8-A134 | NA | NA | -0.0383 | -0.3087 |
| TCGA-C8-A1HJ | NA | NA | NA | NA |
| TCGA-C8-A26X | NA | NA | NA | NA |
| TCGA-C8-A26Y | NA | NA | NA | NA |
| TCGA-C8-A27B | NA | NA | NA | NA |
| TCGA-D8-A13Z | NA | NA | NA | NA |
| TCGA-D8-A142 | NA | NA | 0.2054 | 1.0058 |
| TCGA-D8-A143 | NA | NA | NA | NA |
| TCGA-D8-A147 | NA | NA | NA | NA |
| TCGA-D8-A1JF | NA | NA | NA | NA |
| TCGA-D8-A1JG | NA | NA | NA | NA |
| TCGA-D8-A1JL | NA | NA | NA | NA |
| TCGA-D8-A1XK | NA | NA | NA | NA |
| TCGA-D8-A1XQ | NA | NA | NA | NA |
| TCGA-D8-A27F | NA | NA | NA | NA |
| TCGA-D8-A27H | NA | NA | NA | NA |
| TCGA-D8-A27M | NA | NA | NA | NA |
| TCGA-E2-A14N | NA | NA | NA | NA |
| TCGA-E2-A14R | NA | NA | NA | NA |
| TCGA-E2-A14X | NA | NA | NA | NA |
| TCGA-E2-A150 | NA | NA | 1.1696 | -0.2715 |
| TCGA-E2-A158 | NA | NA | 0.6638 | 0.1528 |
| TCGA-E2-A159 | NA | NA | 0.2508 | -1.9838 |
| TCGA-E2-A1AZ | NA | NA | NA | NA |
| TCGA-E2-A1B6 | NA | NA | NA | NA |
| TCGA-E2-A1L7 | NA | NA | NA | NA |
| TCGA-E2-A1LG | NA | NA | NA | NA |
| TCGA-E2-A1LH | NA | NA | NA | NA |
| TCGA-E2-A1LI | NA | NA | NA | NA |
| TCGA-E2-A1LK | NA | NA | NA | NA |
| TCGA-E2-A1LL | NA | NA | NA | NA |
| TCGA-E2-A1LS | NA | NA | NA | NA |
| TCGA-E9-A1ND | NA | NA | NA | NA |
| TCGA-E9-A22G | NA | NA | NA | NA |
| TCGA-EW-A1OV | NA | NA | NA | NA |
| TCGA-EW-A1OW | NA | NA | NA | NA |
| TCGA-EW-A1P1 | NA | NA | NA | NA |
| TCGA-EW-A1P4 | NA | NA | NA | NA |
| TCGA-EW-A1P7 | NA | NA | NA | NA |
| TCGA-EW-A1P8 | NA | NA | NA | NA |
| TCGA-EW-A1PB | NA | NA | NA | NA |

**SUPPLEMENTARY TABLE 5.** Clinico-pathologic characteristics of breast cancer patients used for IHC analysis

| No. | ER* | PR* | p53 | HER2 | Histologic grade** | Tumor size | Follow-up months | Follow-up result | Cause of death |
| --- | --- | --- | --- | --- | --- | --- | --- | --- | --- |
| 1 | + | + | low expression | 1+ | Ⅱ | 3.0 cm | 84 | alive |  |
| 2 | + | + | low expression | 0 | Ⅱ | 3.5 cm | 84 | alive |  |
| 3 | - | - | no expression | 0 | Ⅱ | 4.2 cm | 84 | alive |  |
| 4 | + | + | low expression | 0 | Ⅲ | 3.0 cm | 34 | dead | cancer |
| 5 | - | - | no expression | 0 | Ⅲ | 2.5 cm | 84 | alive |  |
| 6 | + | - | over expression | 0 | Ⅱ | 2.5 cm | 83 | alive |  |
| 7 | + | - | over expression | 0 | Ⅱ | 10.0 cm | 57 | dead | cancer |
| 8 | + | - | low expression | 2+ | Ⅱ | 6.0 cm | 82 | alive |  |
| 9 | - | - | over expression | 0 | Ⅲ | 2.5 cm | 82 | alive |  |
| 10 | - | - | over expression | 3+ | Ⅱ | 4.5 cm | 82 | alive |  |
| 11 | - | - | no expression | 3+ | Ⅲ | 2.5 cm | 82 | alive |  |
| 12 | + | + | no expression | 0 | Ⅲ | 3.0 cm | 80 | alive |  |
| 13 | - | - | over expression | 0 | Ⅲ | 2.5 cm | 79 | alive |  |
| 14 | - | - | over expression | 0 | Ⅲ | 5.0 cm | 38 | dead | cancer |
| 15 | + | + | low expression | 2+ | Ⅲ | 5.2 cm | 79 | alive |  |
| 16 | + | + | no expression | 0 | Ⅱ | 2.2 cm | 78 | alive |  |
| 17 | + | - | low expression | 1+ | Ⅱ | 2.5 cm | 78 | alive |  |
| 18 | + | - | low expression | 3+ | Ⅱ | 2.5 cm | 165 | dead |  |
| 19 | - | - | over expression | 0 | Ⅲ | 5.5 cm | 77 | alive |  |
| 20 | + | - | over expression | 0 | Ⅱ | 5.0 cm | 238 | alive |  |
| 21 | + | + | low expression | 1+ | Ⅲ | 4.5 cm | 77 | alive |  |
| 22 | - | - | no expression | 0 | uk | 3.5 cm | 77 | alive |  |
| 23 | + | - | low expression | 0 | Ⅱ | 3.0 cm | 76 | alive |  |
| 24 | - | - | over expression | 0 | Ⅱ | 3.0 cm | 76 | alive |  |
| 25 | + | - | low expression | 1+ | Ⅱ | 5.6 cm | 247 | dead |  |
| 26 | + | - | no expression | 3+ | Ⅲ | 4.0 cm | 76 | alive |  |
| 27 | - | - | low expression | 3+ | Ⅲ | 3.0 cm | 31 | dead |  |
| 28 | + | - | low expression | 0 | Ⅱ | 5.5 cm | 75 | alive |  |
| 29 | + | - | low expression | 0 | Ⅱ | 5.0 cm | 75 | alive |  |
| 30 | - | - | no expression | 3+ | Ⅱ | 10.0 cm | 59 | dead | cancer |
| 31 | - | - | over expression | 0 | Ⅲ | 3.5 cm | 9 | dead | cancer |
| 32 | - | - | no expression | 0 | Ⅲ | 3.0 cm | 82 | alive |  |
| 33 | - | - | over expression | 0 | Ⅲ | 2.5 cm | 81 | alive |  |
| 34 | + | - | over expression | 3+ | Ⅲ | 3.5 cm | 9 | dead | cancer |
| 35 | - | - | low expression | 3+ | Ⅲ | 4.5 cm | 78 | alive |  |
| 36 | - | - | no expression | 0 | Ⅲ | 5.0 cm | 75 | alive |  |
| 37 | - | - | over expression | 0 | Ⅲ | 3.0 cm | 73 | alive |  |
| 38 | - | - | no expression | 3+ | Ⅲ | 3.5 cm | 72 | alive |  |
| 39 | + | - | over expression | 3+ | Ⅱ | 6.0 cm | 72 | alive |  |
| 40 | - | - | over expression | 0 | Ⅱ | 10.0 cm | 33 | dead | cancer |
| 41 | - | - | over expression | 0 |  |  |  |  |  |
| 42 | - | - | no expression | 0 |  |  |  |  |  |
| 43 | - | - | over expression | 0 |  |  |  |  |  |
| 44 | + | - | over expression | 3+ |  |  |  |  |  |
| 45 | - | - | low expression | 3+ |  |  |  |  |  |
| 46 | - | - | no expression | 0 |  |  |  |  |  |
| 47 | - | - | over expression | 0 |  |  |  |  |  |
| 48 | - | - | no expression | 3+ |  |  |  |  |  |
| 49 | + | - | over expression | 3+ |  |  |  |  |  |
| 50 | - | - | over expression | 0 |  |  |  |  |  |

**SUPPLEMENTARY TABLE 6. Treatment profile of TCGA-TNBC patients**

| Patient | Adjuvant |
| --- | --- |
| TCGA-A1-A0SK | Re-excision |
| TCGA-A7-A26I | Radiation + pharmaceutical therapy + chemo |
| TCGA-A7-A26F | Radiation + pharmaceutical therapy + chemo |
| TCGA-A2-A0SX | Radiation + pharmaceutical therapy + chemo |
| TCGA-EW-A1P7 | Radiation + pharmaceutical therapy + chemo |
| TCGA-E2-A1LG | Radiation + pharmaceutical therapy + chemo |
| TCGA-E2-A158 | Radiation + pharmaceutical therapy + chemo |
| TCGA-E2-A14R | Radiation + pharmaceutical therapy + chemo |
| TCGA-EW-A1PB | Radiation + pharmaceutical therapy |
| TCGA-E2-A1LK | Radiation + pharmaceutical therapy |
| TCGA-D8-A1XQ | Radiation + pharmaceutical therapy |
| TCGA-D8-A1JL | Radiation + pharmaceutical therapy |
| TCGA-D8-A1JF | Radiation + pharmaceutical therapy |
| TCGA-C8-A26Y | Radiation + pharmaceutical therapy |
| TCGA-C8-A12V | Radiation + pharmaceutical therapy |
| TCGA-BH-A18G | Radiation + pharmaceutical therapy |
| TCGA-BH-A0WA | Radiation + pharmaceutical therapy |
| TCGA-BH-A0BG | Radiation + pharmaceutical therapy |
| TCGA-B6-A0WX | Radiation + pharmaceutical therapy |
| TCGA-B6-A0RU | Radiation + pharmaceutical therapy |
| TCGA-B6-A0RT | Radiation + pharmaceutical therapy |
| TCGA-B6-A0RS | Radiation + pharmaceutical therapy |
| TCGA-B6-A0RN | Radiation + pharmaceutical therapy |
| TCGA-B6-A0RG | Radiation + pharmaceutical therapy |
| TCGA-B6-A0RE | Radiation + pharmaceutical therapy |
| TCGA-B6-A0IQ | Radiation + pharmaceutical therapy |
| TCGA-AR-A0U0 | Radiation + pharmaceutical therapy |
| TCGA-AN-A0XU | Radiation + pharmaceutical therapy |
| TCGA-AN-A0G0 | Radiation + pharmaceutical therapy |
| TCGA-AN-A0FX | Radiation + pharmaceutical therapy |
| TCGA-AN-A0FL | Radiation + pharmaceutical therapy |
| TCGA-AN-A0AT | Radiation + pharmaceutical therapy |
| TCGA-AN-A0AR | Radiation + pharmaceutical therapy |
| TCGA-AN-A0AL | Radiation + pharmaceutical therapy |
| TCGA-AN-A04D | Radiation + pharmaceutical therapy |
| TCGA-A2-A0YM | Radiation + pharmaceutical therapy |
| TCGA-A2-A0ST | Radiation + hormone therapy + chemo |
| TCGA-E2-A1L7 | Radiation + hormone + chemo + Pharmaceutical Therapy |
| TCGA-E9-A1ND | Radiation + hormone |
| TCGA-AO-A0JL | Radiation + chemo + Targeted Molecular Therapy |
| TCGA-E2-A14X | Radiation + chemo + Bisphosphonate Therapy |
| TCGA-EW-A1P8 | Radiation + chemo |
| TCGA-EW-A1P4 | Radiation + chemo |
| TCGA-EW-A1P1 | Radiation + chemo |
| TCGA-EW-A1OW | Radiation + chemo |
| TCGA-EW-A1OV | Radiation + chemo |
| TCGA-E9-A22G | Radiation + chemo |
| TCGA-E2-A1LS | Radiation + chemo |
| TCGA-E2-A1LL | Radiation + chemo |
| TCGA-E2-A1LI | Radiation + chemo |
| TCGA-E2-A1LH | Radiation + chemo |
| TCGA-E2-A1B6 | Radiation + chemo |
| TCGA-E2-A1AZ | Radiation + chemo |
| TCGA-E2-A159 | Radiation + chemo |
| TCGA-E2-A150 | Radiation + chemo |
| TCGA-E2-A14N | Radiation + chemo |
| TCGA-D8-A27M | Radiation + chemo |
| TCGA-D8-A27H | Radiation + chemo |
| TCGA-D8-A27F | Radiation + chemo |
| TCGA-D8-A1XK | Radiation + chemo |
| TCGA-D8-A1JG | Radiation + chemo |
| TCGA-D8-A147 | Radiation + chemo |
| TCGA-D8-A143 | Radiation + chemo |
| TCGA-D8-A142 | Radiation + chemo |
| TCGA-C8-A27B | Radiation + chemo |
| TCGA-C8-A26X | Radiation + chemo |
| TCGA-C8-A134 | Radiation + chemo |
| TCGA-C8-A131 | Radiation + chemo |
| TCGA-BH-A0RX | Radiation + chemo |
| TCGA-BH-A0E6 | Radiation + chemo |
| TCGA-BH-A0E0 | Radiation + chemo |
| TCGA-BH-A0BW | Radiation + chemo |
| TCGA-BH-A0BL | Radiation + chemo |
| TCGA-BH-A0B9 | Radiation + chemo |
| TCGA-BH-A0B3 | Radiation + chemo |
| TCGA-BH-A0AV | Radiation + chemo |
| TCGA-AR-A256 | Radiation + chemo |
| TCGA-AR-A1AY | Radiation + chemo |
| TCGA-AR-A1AR | Radiation + chemo |
| TCGA-AR-A1AQ | Radiation + chemo |
| TCGA-AR-A1AI | Radiation + chemo |
| TCGA-AR-A0U4 | Radiation + chemo |
| TCGA-AR-A0U1 | Radiation + chemo |
| TCGA-AR-A0TU | Radiation + chemo |
| TCGA-AR-A0TS | Radiation + chemo |
| TCGA-AQ-A04J | Radiation + chemo |
| TCGA-AO-A12F | Radiation + chemo |
| TCGA-AO-A129 | Radiation + chemo |
| TCGA-AO-A128 | Radiation + chemo |
| TCGA-AO-A124 | Radiation + chemo |
| TCGA-AO-A0J6 | Radiation + chemo |
| TCGA-AO-A0J4 | Radiation + chemo |
| TCGA-AO-A0J2 | Radiation + chemo |
| TCGA-AO-A03U | Radiation + chemo |
| TCGA-A8-A07O | Radiation + chemo |
| TCGA-A8-A07C | Radiation + chemo |
| TCGA-A7-A26G | Radiation + chemo |
| TCGA-A7-A0DA | Radiation + chemo |
| TCGA-A7-A0CE | Radiation + chemo |
| TCGA-A2-A1G6 | Radiation + chemo |
| TCGA-A2-A0YE | Radiation + chemo |
| TCGA-A2-A0T2 | Radiation + chemo |
| TCGA-A2-A0T0 | Radiation + chemo |
| TCGA-A2-A0D2 | Radiation + chemo |
| TCGA-A2-A0D0 | Radiation + chemo |
| TCGA-A2-A04T | Radiation + chemo |
| TCGA-A2-A04Q | Radiation + chemo |
| TCGA-A1-A0SP | Radiation + chemo |
| TCGA-A1-A0SO | Radiation + chemo |
| TCGA-A2-A0CM | Radiation + pharmaceutical + Bisphosphonate Therapy + chemo + hormone therapy |
| TCGA-A2-A04U | Radiation + Bisphosphonate Therapy + chemo |
| TCGA-D8-A13Z | Radiation + hormone + chemo |
| TCGA-C8-A1HJ | NA |
| TCGA-BH-A1FC | NA |
| TCGA-BH-A1EW | NA |
| TCGA-BH-A18V | NA |
| TCGA-BH-A18T | NA |
| TCGA-BH-A18Q | NA |
| TCGA-B6-A0IK | NA |
| TCGA-B6-A0IE | NA |
| TCGA-A8-A09X | NA |
| TCGA-A8-A08R | NA |
| TCGA-A2-A04P | NA |

**SUPPLEMENTARY TABLE 7.** List of TNBC cell lines from Genomics of Drug Sensitivity database used for the analysis of mTOR inhibitor treatment response

| **Cells treated with Rapamycin and their IC50 values (µM)** | | **Cells treated with Temsirolimus and their IC50 values (µM)** | |
| --- | --- | --- | --- |
| **TNBC cells (n= 22)** | **Rapamycin IC50** | **TNBC cells (n= 21)** | **Temsirolimus IC50** |
| BT-20  BT-549  CAL-120  CAL-148  CAL-51  CAL-85-1  DU-4475  HCC1143  HCC1187  HCC1395  HCC1599  HCC1937  HCC2157  HCC38  HCC70  HDQ-P1  Hs-578-T  MDA-MB-157  MDA-MB-231  MDA-MB-436  MDA-MB-453  MDA-MB-468 | 0.245898  0.049629  0.039912  0.003599  0.021304  0.499309  0.102134  0.110335  0.308749  0.100889  1.073832  0.16478  0.056727  0.355978  0.030572  0.153629  0.020733  0.483608  0.036851  0.241656  0.006085  0.018583 | BT-20  BT-549  CAL-120  CAL-51  CAL-85-1  DU-4475  HCC1143  HCC1187  HCC1395  HCC1599  HCC1937  HCC2157  HCC38  HCC70  HDQ-P1  Hs-578-T  MDA-MB-157  MDA-MB-231  MDA-MB-436  MDA-MB-453  MDA-MB-468 | 5.949025  3.912617  4.310597  1.788552  0.497792  2.615341  6.614247  20.457984  2.507105  37.321061  6.27956  1.378903  47.661037  1.194201  3.91254  0.273646  11.50382  2.229208  8.113194  0.14953  1.540286 |

**SUPPLEMENTARY TABLE 8.** Cell viabilities of mTOR inhibitors treated TNBC cells

| **Treatment** | **HCC1937** | | | **Hs578T** | | |
| --- | --- | --- | --- | --- | --- | --- |
| **Rapamycin Conc. (µM)** | **Replicate1** | **Replicate2** | **Replicate3** | **Replicate1** | **Replicate2** | **Replicate3** |
| 100  50  25  12.5  6.25  3.125  0 | 49%  75%  98%  104%  101%  102%  100% | 49%  62%  80%  86%  85%  79%  100% | 36%  62%  85%  84%  84%  84%  100% | 25%  65%  68%  71%  67%  69%  100% | 25%  63%  67%  64%  62%  68%  100% | 25%  58%  69%  74%  68%  70%  100% |
| **IC50** | **97.37** | **94.81** | **68.85** | **64.84** | **63.38** | **59.15** |
| **Temsirolimus Conc. (µM)** | **Replicate1** | **Replicate2** | **Replicate3** | **Replicate1** | **Replicate2** | **Replicate3** |
| 100  50  25  12.5  6.25  3.125  0 | 27%  58%  47%  80%  90%  79%  100% | 25%  69%  66%  93%  94%  86%  100% | 10%  27%  78%  80%  111%  123%  100% | 9%  11%  53%  82%  82%  75%  100% | 8%  9%  43%  75%  76%  72%  100% | 12%  24%  57%  70%  78%  65%  100% |
| **IC50** | **59.79** | **67.45** | **36.57** | **26.27** | **21.48** | **28.96** |
| **Everolimus Conc. (µM)** | **Replicate1** | **Replicate2** | **Replicate3** | **Replicate1** | **Replicate2** | **Replicate3** |
| 100  50  25  12.5  6.25  3.125  0 | 7%  13%  81%  83%  91%  96%  100% | 8%  10%  68%  82%  86%  90%  100% | 8%  9%  70%  83%  84%  87%  100% | 7%  7%  27%  59%  64%  64%  100% | 9%  13%  34%  82%  81%  89%  100% | 9%  11%  29%  70%  71%  72%  100% |
| **IC50** | **34.29** | **31.00** | **31.39** | **15.19** | **19.84** | **17.53** |


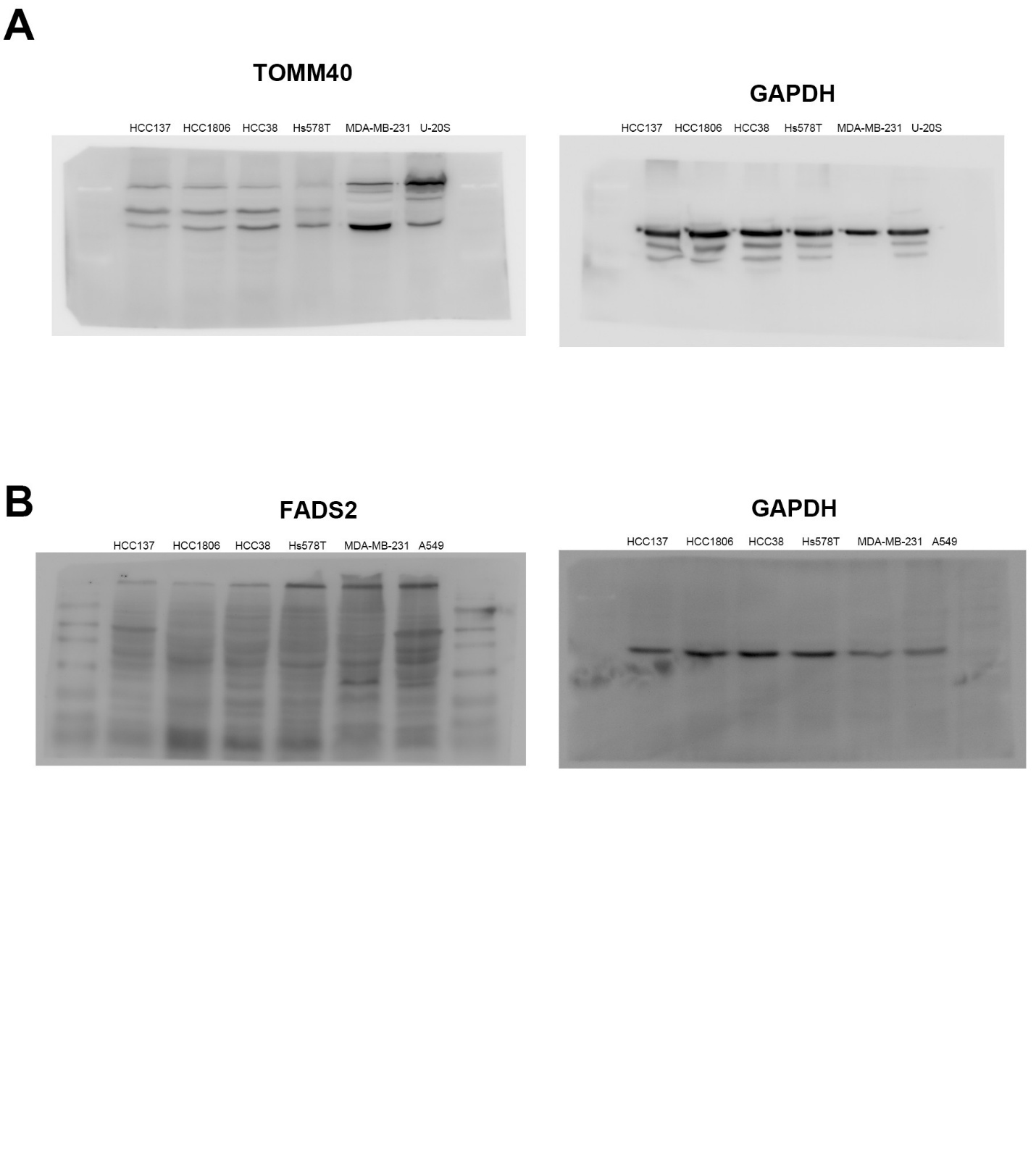


**Fig S1.** Originally uncut blots from Western blot analyses for TOMM40 (**A**) and FADS2 (**B**) with their respective GAPDH (**Right**) in TNBC cell lines HCC1937, HCC1806, HCC38, Hs578T, MDA-MB-231 and positive controls A549 and U-20S.


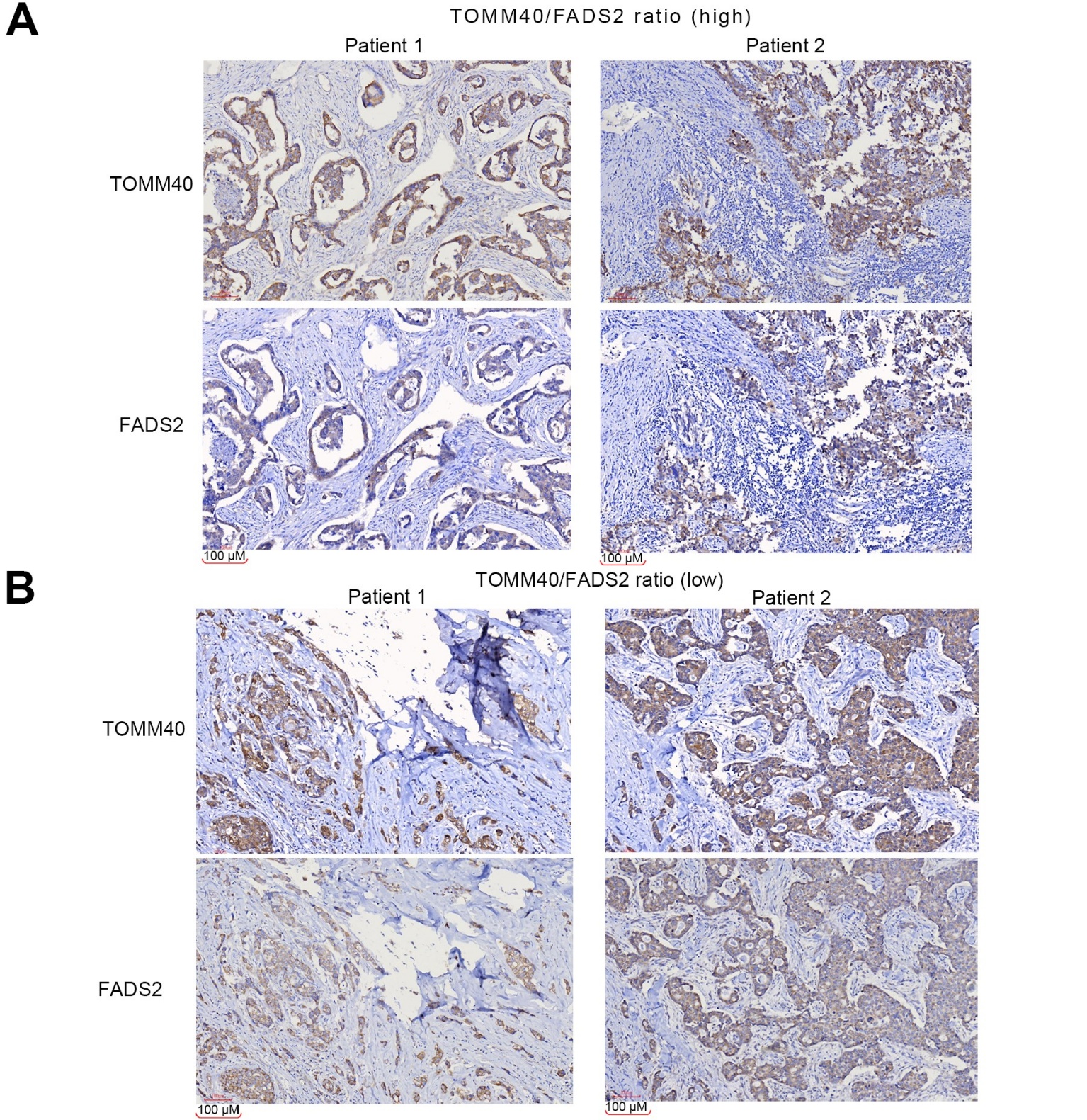


**Fig S2.** The representatives of high (**A**) and low (**B**) TOMM40/FADS2 ratios in IHC staining against breast cancer tissues.


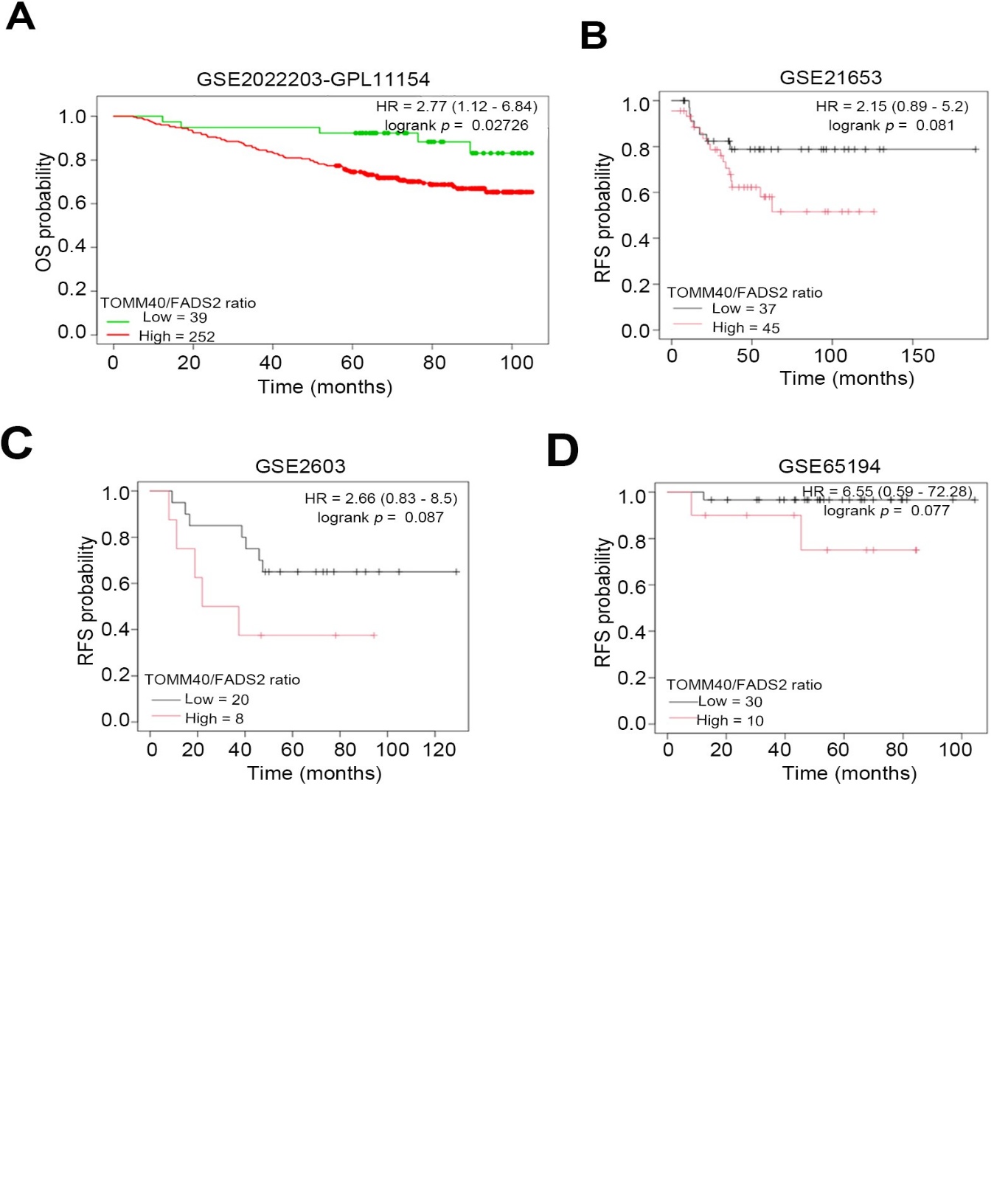


**Fig S3.** (A-D) Kaplan-Meier analyses for the TOMM40/FADS2 expression ratio using overall survival (OS) in GSE2022203-GPL11154 (A) and recurrence-free survival (RFS) in GSE21653 (B) GSE2603 (C) and GSE65194 (D) conditions under a minimized p-values against GEO TNBC patients.


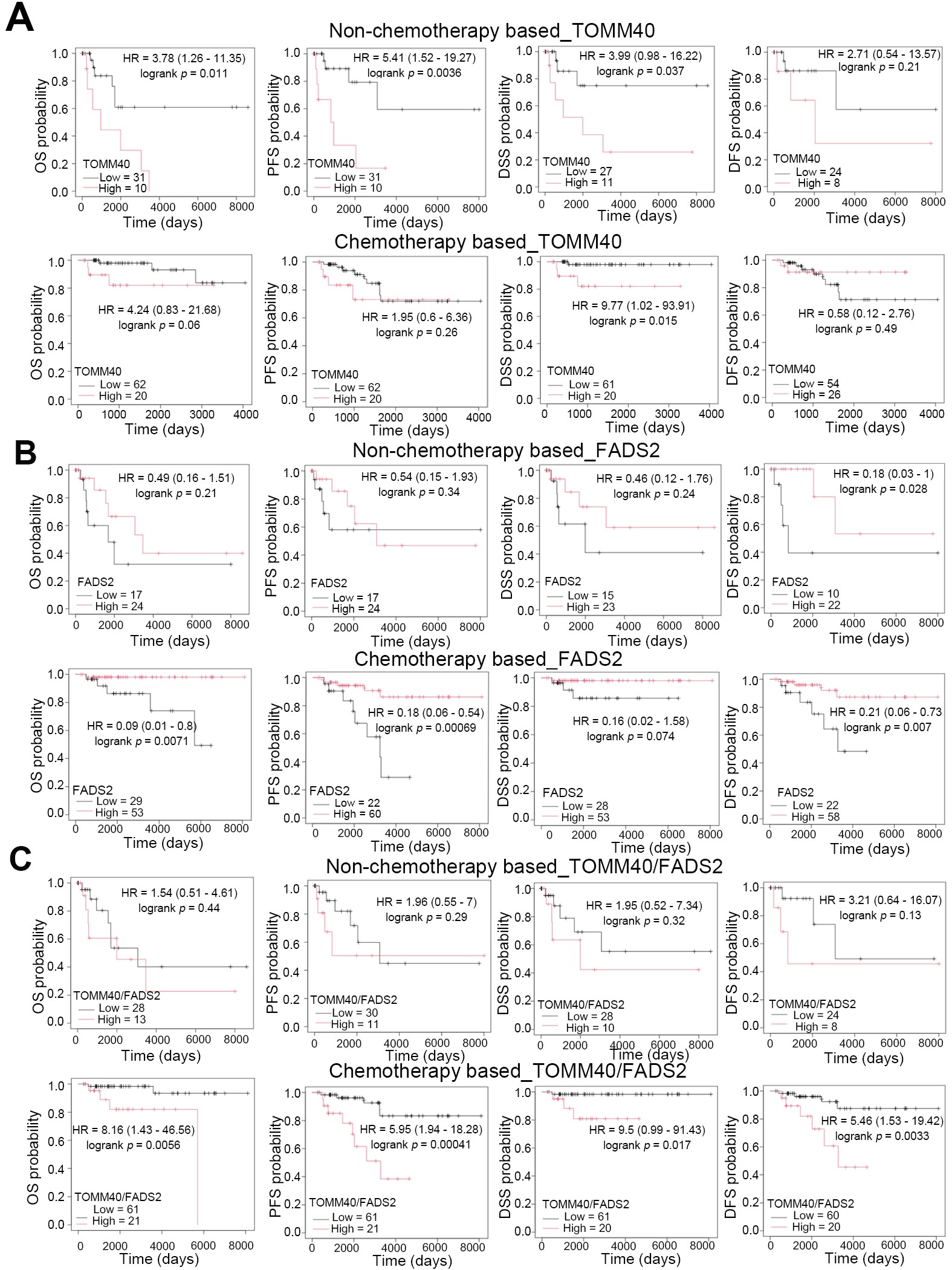


**Fig S4.** (A-C) Kaplan-Meier analyses for the TOMM40 (A), FADS2 (B) and TOMM40/FADS2 expression ratio (C) in non-chemotherapy based (upper) and chemotherapy based treatment (lower) using overall survival (OS), progression-free index (PFI), disease-specific survival (DSS) and disease-free survival (DFS) conditions under a minimized p-values against TCGA TNBC patients.
